# Expanding the boundaries of PTE: successful integration with multiple major cardiac procedures

**DOI:** 10.1093/jscr/rjaf896

**Published:** 2026-02-05

**Authors:** Emily Hay-Arthur, Elizabeth J Bashian, Sarah Y Park, Jordan R H Hoffman, Michael T Cain

**Affiliations:** Division of Cardiothoracic Surgery, University of Colorado Anschutz Medical Campus, 12631 East 17th Avenue, Aurora, CO 80045, United States; Division of Cardiothoracic Surgery, University of Colorado Anschutz Medical Campus, 12631 East 17th Avenue, Aurora, CO 80045, United States; Division of Cardiothoracic Surgery, University of Colorado Anschutz Medical Campus, 12631 East 17th Avenue, Aurora, CO 80045, United States; Division of Cardiothoracic Surgery, University of Colorado Anschutz Medical Campus, 12631 East 17th Avenue, Aurora, CO 80045, United States; Division of Cardiothoracic Surgery, University of Colorado Anschutz Medical Campus, 12631 East 17th Avenue, Aurora, CO 80045, United States

**Keywords:** pulmonary thromboendarterectomy, pulmonary hypertension, coronary artery bypass, cardiac surgical procedures, combined modality therapy

## Abstract

Pulmonary thromboendarterectomy (PTE) is the only curative treatment for chronic thromboembolic pulmonary hypertension. While combining PTE with a single cardiac procedure is well established, evidence for performing it with multiple major procedures is limited. We report a successful bilateral PTE with coronary artery bypass grafting, ascending aortic and hemiarch repair, patent foramen ovale closure, and left atrial appendage ligation. The patient recovered uneventfully, with marked functional and hemodynamic improvement at 6 months. This case demonstrates the safety and feasibility of complex cardiac surgery with PTE, underscoring the value of thorough, multidisciplinary preoperative planning.

## Introduction

Chronic thromboembolic pulmonary hypertension (CTEPH) is an obstructive, fibrotic transformation of the pulmonary vasculature occurring in about 4% of patients after pulmonary embolism. It is defined as pulmonary artery pressure >20 mmHg, pulmonary vascular resistance >3 Woods units, and pulmonary capillary wedge pressure <15 mmHg. Untreated, CTEPH causes pulmonary vascular remodeling and the chronically elevated vascular pulmonary vascular resistance predisposes patients to right heart failure, and death [1, 2].

Pulmonary thromboendarterectomy (PTE), the only curative treatment for CTEPH, offering significant survival benefit [3, 4]. Previous reports show PTE can be performed with single additional procedures such as coronary artery bypass grafting (CABG) or valve interventions and literature supports combining multiple cardiac operations [3, 5, 6]. However, no prior reports describe bilateral PTE along with several major cardiac procedures in a single index operation. We describe a patient presenting with CTEPH also in need of aortic valve replacement, CABG, aortic aneurysm repair, patent foramen ovale (PFO) closure, and left atrial appendage ligation (LAAL).

## Case report

**Figure 1:**
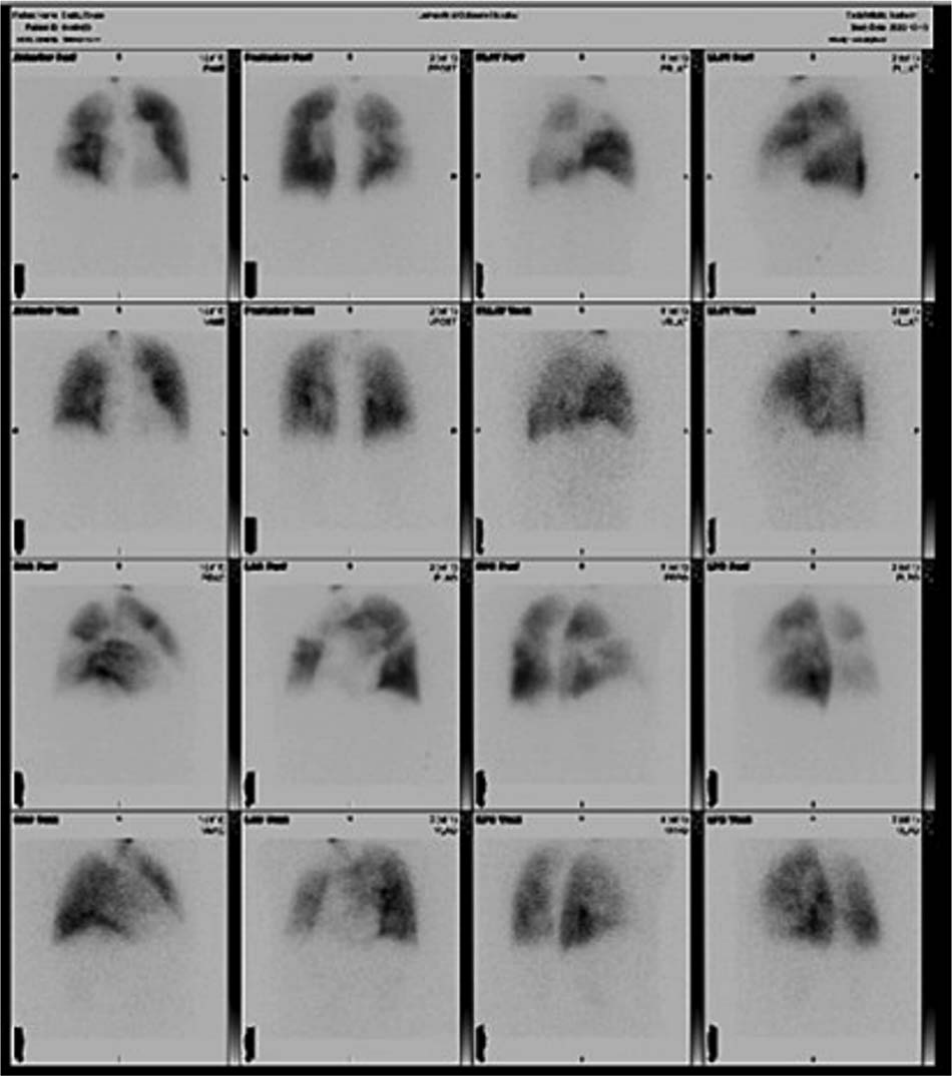
Preoperative ventilation-perfusion (V/Q) scan demonstrating bilateral segmental and subsegmental perfusion defects consistent with chronic thromboembolic pulmonary hypertension.

Informed written consent was obtained. A 68-year-old male with history of CTEPH, chronic obstructive pulmonary disease, coronary artery disease, chronic osteomyelitis, Type II diabetes, and persistent atrial fibrillation presented with worsening dyspnea requiring home oxygen. Preoperative evaluation showed mean pulmonary artery pressure 42 mmHg, PCWP 11 mmHg, PVR 8.66 Wood units, and bilateral mismatches on ventilation–perfusion (V/Q) scan (Fig. 1). Coronary angiogram revealed multivessel disease. Echocardiogram showed severe eccentric aortic insufficiency, ascending aortic aneurysm, PFO, and enlarged right ventricle (RVSP 77 mmHg). Multidisciplinary review deemed the patient suitable for PTE with concomitant procedures.

**Figure 2:**
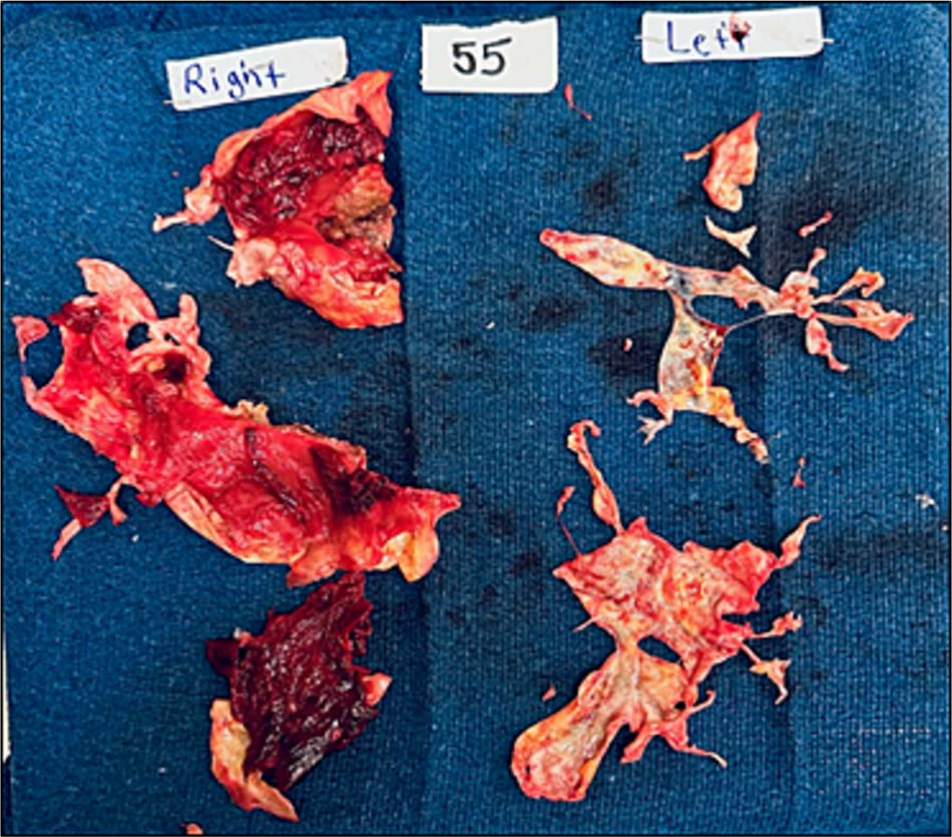
Intraoperative specimen showing large clot burden removed during bilateral pulmonary thromboendartectomy, with both subacute and chronic organized thromboembolic material evident.

**Figure 3:**
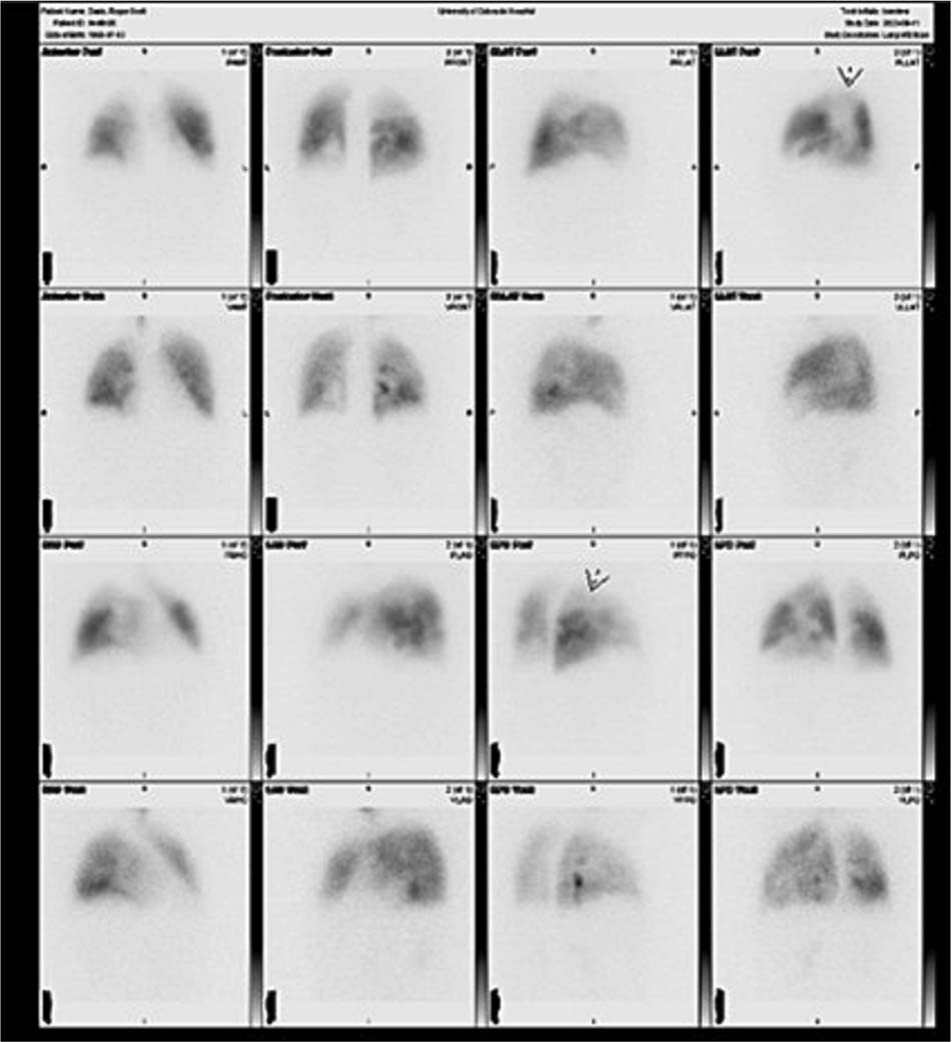
Six-month postoperative V/Q scan demonstrating interval improvement in bilateral pulmonary perfusion, with homogenous distribution of radiotracer and normalization of hilar and vascular markings.

Under general anesthesia, a pulmonary artery catheter and transesophageal echocardiography confirmed preoperative findings. CABG was performed with left internal mammary artery to the left anterior descending artery and saphenous vein graft to the right coronary artery. Bilateral pulmonary endarterectomy with right pulmonary artery patch angioplasty was completed. Disease was Jameson class I on the right and class II on the left (Fig. 2). The patient then underwent aortic valve replacement with an Edwards Inspiris tissue valve, ascending aorta repair, PFO closure, and LAAL. No intraoperative complications occurred. He was extubated on postoperative day 1. Echocardiogram showed a mildly enlarged right ventricle with normal pressures. Mean PA pressures were 24, 18, 24, and 15 mmHg on postoperative days 1–4. He was discharged on day 8 requiring 3 L oxygen at rest and 8 L with exertion. At 6 months, he required no supplemental oxygen and reported improved exercise tolerance. Follow-up V/Q scan showed markedly improved bilateral perfusion (Fig. 3).

## Discussion

PTE remains the gold standard for CTEPH, offering long-term survival benefits not matched by medical therapy [1–3]. Preoperative workup includes echocardiogram, right heart catheterization with pulmonary angiography, V/Q scan, and CTA chest to confirm diagnosis and exclude alternative causes of pulmonary hypertension. Evaluation for concurrent cardiac disease is essential; coronary angiography is indicated in patients with coronary risk factors [7]. Risk models such as STS and EuroSCORE may underestimate complexity in combined PTE cases.

Previous reports support combining PTE with one additional cardiac procedure [5, 6]. Thistlewaite *et al*. reported similar mortality between isolated PTE and PTE with concomitant CABG, PFO closure, or valve surgery [5]. Erdem *et al*. likewise reported good outcomes with combined procedures, most often CABG [6].

Our case extends these findings, showing safe performance of bilateral PTE with CABG, aortic valve replacement, aortic aneurysm repair, PFO closure, and LAAL in a single operation. Success relied on meticulous preoperative planning to minimize ischemic and bypass times. The hemiarch was resected during the first circulatory arrest before addressing the pulmonary arteries, improving exposure. CABG was performed during cooling, and valve/aortic work during rewarming, reducing idle time. This strategy resulted in cross-clamp and bypass times only slightly longer than for PTE alone [8].

Combining multiple procedures avoids staged operations, reducing the risk of repeat sternotomy and ongoing exposure to untreated pulmonary hypertension. Published data suggest no increased risk when adding general cardiac procedures to an aortic case, and concomitant procedures may confer a survival benefit.

## Data Availability

All data generated or analyzed during this study are included in this published article.
